# Integration of mRNA and miRNA Analysis Reveals the Molecular Mechanism Underlying Salt and Alkali Stress Tolerance in Tobacco

**DOI:** 10.3390/ijms20102391

**Published:** 2019-05-14

**Authors:** Jiayang Xu, Qiansi Chen, Pingping Liu, Wei Jia, Zheng Chen, Zicheng Xu

**Affiliations:** 1 National Tobacco Cultivation and Physiology and Biochemistry Research Center, College of Tobacco Science, Henan Agricultural University, Zhengzhou 450002, China; jiayangxu@126.com (J.X.); zchen1004@126.com (Z.C.); 2 Zhengzhou Tobacco Research Institute, Zhengzhou 450001, China; chen_qiansi@163.com (Q.C.); Liu_pingping2012@163.com (P.L.); 3 College of Resources and Environment, Huazhong Agricultural University, Wuhan 430070, China; shuhan_jia@163.com

**Keywords:** salt, alkali, transcriptome, miRNA, tobacco

## Abstract

Salinity is one of the most severe forms of abiotic stress and affects crop yields worldwide. Plants respond to salinity stress via a sophisticated mechanism at the physiological, transcriptional and metabolic levels. However, the molecular regulatory networks involved in salt and alkali tolerance have not yet been elucidated. We developed an RNA-seq technique to perform mRNA and small RNA (sRNA) sequencing of plants under salt (NaCl) and alkali (NaHCO_3_) stress in tobacco. Overall, 8064 differentially expressed genes (DEGs) and 33 differentially expressed microRNAs (DE miRNAs) were identified in response to salt and alkali stress. A total of 1578 overlapping DEGs, which exhibit the same expression patterns and are involved in ion channel, aquaporin (AQP) and antioxidant activities, were identified. Furthermore, genes involved in several biological processes, such as “photosynthesis” and “starch and sucrose metabolism,” were specifically enriched under NaHCO_3_ treatment. We also identified 15 and 22 miRNAs that were differentially expressed in response to NaCl and NaHCO_3_, respectively. Analysis of inverse correlations between miRNAs and target mRNAs revealed 26 mRNA-miRNA interactions under NaCl treatment and 139 mRNA-miRNA interactions under NaHCO_3_ treatment. This study provides new insights into the molecular mechanisms underlying the response of tobacco to salinity stress.

## 1. Introduction

Salinity stress is one of the most constraining environmental factors that threatens both the productivity and quality of crop plants worldwide [1]. In China, a large amount of land is unsuitable for agricultural production because of increasing soil alkalinity. Terrestrial soils affected by salt are divided mainly into saline soils, alkaline soils and salt-alkaline soils [2]. NaCl is a major neutral component that causes salt stress, and NaHCO_3_ and Na_2_CO_3_ play a key role in soil alkalization with increasing pH. By causing osmotic stress, ion toxicity, redox imbalance and metabolic damage, soil salinity and salinization can severely affect biological homeostasis in plants [3,4]. Plants have evolved strategies to cope with salt stress. These strategies include increased accumulation of compatible solutes such as proline, soluble sugars and betaine to adjust to osmotic injury [5]; activation of reactive oxygen species (ROS) scavenging systems to protect plants against oxidative damage triggered by salinity [6]; and regulation of the expression of salt-responsive genes to re-establish ion homeostasis and protect cellular machinery from stress [7,8]. Therefore, to reduce the decrease in crop yield and quality caused by salt stress and promote crop improvement, it would be of great value to understand the molecular mechanism underlying salt tolerance in crop plants.

MicroRNAs (miRNAs) are endogenous, short (18–24 nt), non-coding RNA molecules that negatively regulate gene expression at the post-transcriptional and translational levels [9,10]. A total of 8496 mature miRNAs from 73 plant species have been identified [11]. Many experimental studies have demonstrated that miRNAs play crucial roles in processes associated with plant growth and development, such as cell differentiation [12], organ development [13,14] and flowering [15,16]. In addition to these roles in physiological and metabolic processes, many studies have also shown that miRNAs are widely involved in plant responses to abiotic stress, including drought [17], heat [18], cold [19] and salt stress [20].

Next-generation sequencing (NGS) technology provides a comprehensive understanding of gene status and has become an accurate molecular analytical tool for transcriptional profiling experiments [21]. This sequencing approach has been successfully used to identify the expression of salt-responsive genes in cotton [22], wheat [23], soybean [24], rice [25] and citrus [26]. Moreover, revealing the miRNA-mediated regulatory network of the salt stress response will provide a genetic basis for future studies. A large number of studies have identified many salt stress-related miRNAs in various plant species, such as Arabidopsis [27], wheat [28], cucumber [29], barley [30] and maize [31].

Tobacco (*Nicotiana tabacum* L.) is an important commercial species that is widely cultivated worldwide. Moreover, this plant species is recognized as an excellent model system in plant science for studies on physiological and molecular mechanisms [32,33]. The draft genome sequences of two tobacco species (*N. tabacum* and *Nicotiana benthamiana*) provide a global functional description of identified genes for crop improvement and basic research [34,35]. Although some salt stress-related genes and miRNA profiles have been characterized in tobacco [36,37], our focus here is an integrated analysis of mRNA and miRNA profiling in tobacco under different types of salt and alkali stress.

## 2. Results

### 2.1. mRNA Sequencing Data Analysis

**Figure 1:**
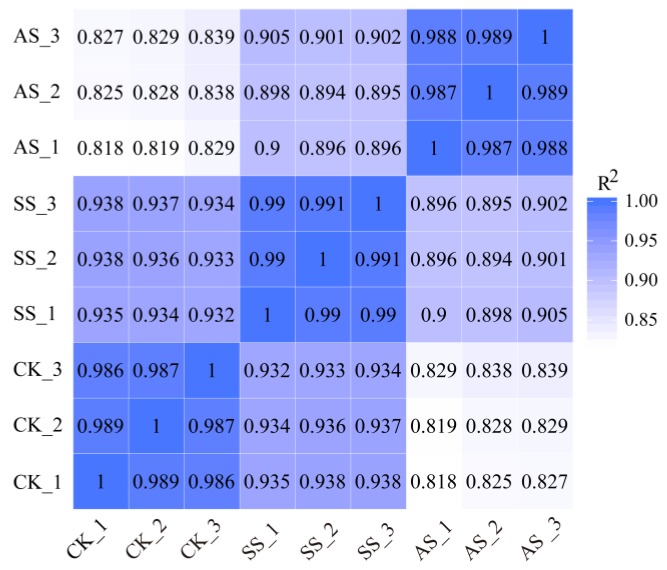
Heatmap of Pearson correlations of the expression levels among samples. CK: control samples, SS: 100 mM NaCl-treated samples, AS: 100 mM NaHCO_3_-treated samples. The numbers in the boxes refer to Pearson’s correlation coefficients (R^2^ values). The number “1” in the boxes represents a high degree of correlation.

A total of nine qualified libraries from the control (CK), NaCl (salt stress, SS) and NaHCO_3_ (alkali stress, AS) treatments, with three replicates per treatment, were sequenced. An overview of the sequencing and assembly data is provided in Table 1. After removal of the low-quality reads from the raw data, approximately 22.14, 26.21 and 26.70 Gb of clean reads were obtained in the CK, SS and AS groups. More than 93.2% of the reads had Q-scores at the Q30 level (an error threshold of less than 0.01%), and more than 93.5% of the clean reads were aligned. To evaluate the gene expression reliability among these replicates, Pearson correlation analysis was conducted. As shown in Figure 1, the results of the correlation analysis indicated that tobacco exhibited more than 85% similarity among the CK, SS and AS treatments.

### 2.2. Differentially Expressed Genes under Salt-Alkali Stress

**Figure 2:**
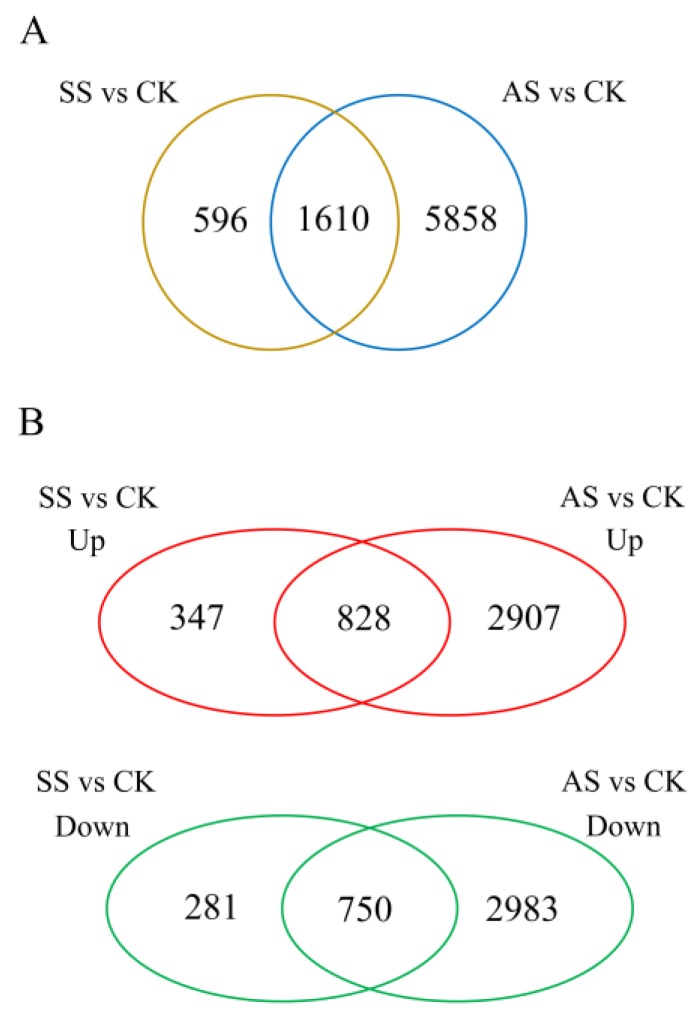
Differential gene expression analysis of the SS vs. CK and AS vs. CK comparisons. (**A**) Venn diagram of overlapping differentially expressed genes (DEGs) among salt-alkali stress comparisons. (**B**) Venn diagrams illustrating the overlap between the upregulated and downregulated genes in the SS vs. CK and AS vs. CK comparisons.

To identify genes that are differentially regulated under various salt-alkali conditions, we used the DESeq software to perform a differential expression analysis between the treatment and CK samples. With strict criteria of |log_2_FC|> 2 and *p*-value less than 0.05 (|log_2_FC|> 2, *p* < 0.05), a total of 2206 genes (Table S1) and 7468 genes (Table S2) were found to be differentially expressed in response to SS and AS, respectively (Figure 2A). There were many more differentially expressed genes (DEGs) in the AS treatment than in the SS treatment, indicating that NaHCO_3_ treatment induced a significantly greater number of biological and metabolic changes than did the NaCl treatment. To better characterize the salt-alkali stress-induced changes in gene expression in tobacco, the overlapping DEGs between the SS vs. CK and AS vs. CK comparable groups are shown as a Venn diagram in Figure 2B. Detailed analysis of these 1610 overlapping DEGs revealed that 1578 of them exhibited the same trends under salt-alkali stress: 828 genes were upregulated and 750 genes were downregulated in both the SS vs. CK and AS vs. CK comparisons.

### 2.3. Functional Classification of Salt-Alkali Responsive Genes

To better understand the functions of the DEGs, we mapped all of these salt-alkali responsive genes to the Gene Ontology (GO) database. In total, we identified 92 and 206 significantly enriched GO terms (false discovery rate (FDR) < 0.05) in the SS and AS treatments, respectively (Table S3). Among these GO terms, some crucial biological processes related to “response to water (GO: 0009415),” “antioxidant activity (GO: 0016209)” and “oxidoreductase activity (GO: 0016491)” were observed in both the SS and AS treatments, while genes involved in “photosystem (GO: 0009521),” “cellular carbohydrate metabolic process (GO: 0044262),” “glucan metabolic process (GO: 0006073)” and “defense response (GO: 0006952)” were enriched in only the AS treatment.

**Figure 3:**
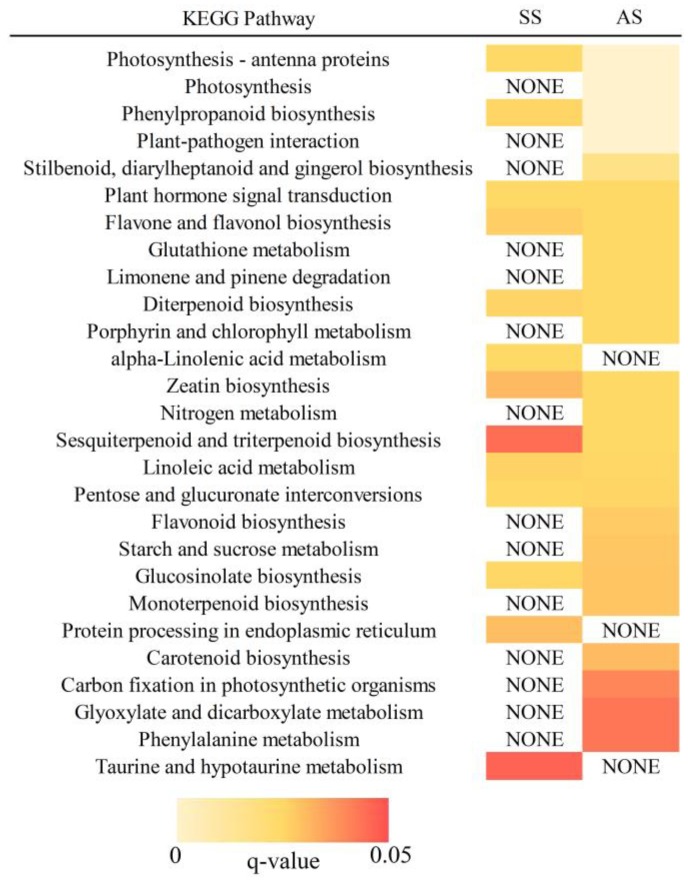
Heatmap of Kyoto Encyclopedia of Genes and Genomes (KEGG) pathway analysis of DEGs in the SS and AS treatments. A total of 13 and 24 enriched KEGG pathways were identified in the SS and AS samples, respectively. The box colour represents the q-value of the pathway.

Kyoto Encyclopedia of Genes and Genomes (KEGG) pathway analysis provided information concerning gene function. A total of 13 and 24 pathways were enriched in the SS and AS treatments, respectively (Figure 3). The enriched KEGG pathways revealed that the DEGs detected under salt-alkali stress were enriched in “photosynthesis-antenna proteins,” “plant hormone signal transduction” and “glucosinolate biosynthesis”. Fourteen KEGG pathways, including “photosynthesis,” “starch and sucrose metabolism,” “glutathione metabolism” and “carbon fixation in photosynthetic organisms,” were specifically enriched in the AS treatment. These results suggest that the DEGs identified in this study may play important roles in salt and alkali stress in tobacco.

### 2.4. miRNA Sequencing Data Analysis

Nine small RNA (sRNA) libraries were constructed with three biological replicates for the CK, SS and AS treatments. A total of 44,642,448, 39,911,768 and 42,157,754 raw reads were obtained from the CK, SS and AS samples, respectively. After the adapter sequences, poly-A sequences, low-quality reads and reads shorter than 18 nt were discarded, 43,327,623, 38,719,988, and 40,868,990 clean reads were obtained from the CK, SS and AS samples, respectively. A majority of the sRNAs were 21–24 nt in length in all nine libraries, with 24 nt being the most frequent length (Figure S1). On the basis of the method described above, a total of 88 mature miRNAs and 102 novel miRNAs were identified in these nine sRNA libraries (Table S4). Details concerning the distribution of the sRNAs are provided in Table 2.

### 2.5. Differential Expression of miRNAs in Response to Salt-Alkali Stress

With respect to the miRNA-seq data analysis, a total of 33 differentially expressed miRNAs (DE miRNAs) were detected in the SS and AS treatments with the criteria |log_2_FC|> 0.5 and *p*-value < 0.05, 16 of which were identified as novel miRNAs (Table 3). Five conserved and 10 novel miRNAs were significantly differentially expressed under SS conditions, four of whose expression was upregulated and 11 of whose was downregulated. Fourteen conserved and eight novel miRNAs were differentially expressed in response to AS, and among those miRNAs, compared with that of the CK, the expression of 13 and nine was upregulated and downregulated, respectively. Of these 33 DE miRNAs, four, namely, nta-miR156a, nta-miR6149a, miR-novel_64 and miR-novel_216, that overlapped between the SS and AS samples were identified. As expected, with the exception of nta-miR156a, all of these overlapping DE miRNAs exhibited similar regulation of expression under the SS and AS treatments.

### 2.6. Integration Analysis of the miRNAs and mRNAs

**Figure 4:**
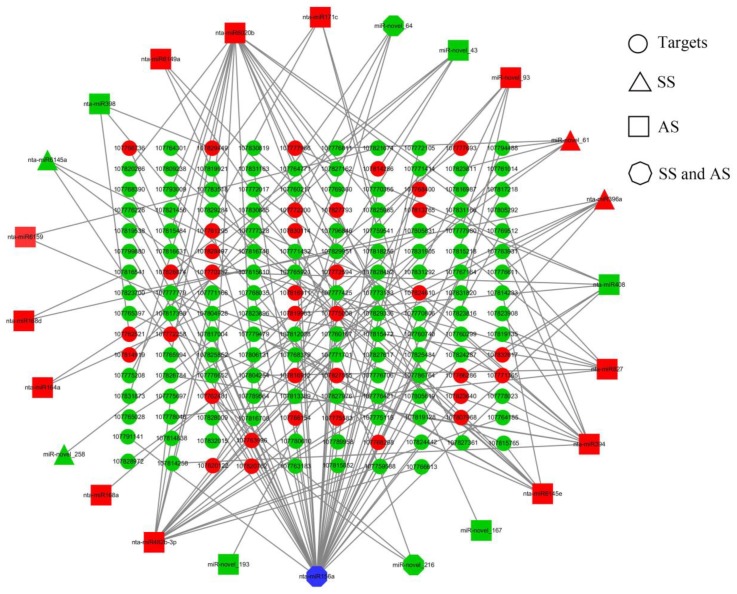
miRNA-mRNA correlation network. The circles in the network indicate the target mRNAs. The triangles, squares and octagons in the network indicate the DE miRNAs regulated by the SS, AS, and SS and AS treatments, respectively. Downregulated mRNAs and miRNAs are shown as green, and upregulated mRNAs and miRNAs are shown as red. Differential expression regulation by both SS and AS is shown in blue.

The interaction between DE miRNAs and their targets was investigated using Cystoscape (version 3.6.1). A total of 165 miRNA-mRNA interactions were identified among the SS and AS treatments, with the involvement of 24 DE miRNAs and 158 DE mRNAs (Figure 4, Table S5). Figure 4 shows that a single miRNA can regulate multiple mRNAs and that a single mRNA can be targeted by more than one miRNA. These results suggest that the miRNA and mRNA interaction network involved in salt and alkali stress is highly complex, especially for different miRNAs with their targets under various conditions.

### 2.7. RT-qPCR Validation of DE mRNAs and miRNAs

**Figure 5:**
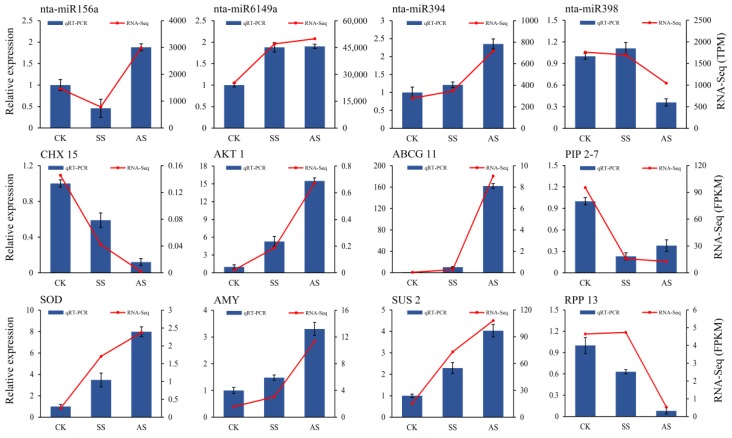
RT-qPCR analysis of miRNAs and mRNAs. The miRNAs and mRNAs were isolated from tobacco treated with NaCl (SS) and NaHCO3 (AS), respectively. The 5.8S rRNA and ef1α genes were chosen as the internal controls for miRNA and mRNA, respectively.

To validate the mRNA and miRNA sequencing data, the expression levels of four DE miRNAs (nta-miR156a, nta-miR6149a, nta-miR394, nta-miR398) and eight DEGs (*CHX 15*, *AKT 1*, *ABCG 11*, *PIP 2-7*, *SOD*, *AMY*, *SUS2*, *RPP 13*), which were determined to be important for salt and alkali stress in our study, were manually selected and analyzed by RT-qPCR (Figure 5). As expected, with the exception of that of RPP 13 in the SS treatment, the expression patterns of most of the examined miRNAs and genes were similar, showing the accuracy and reliability of the RNA-seq data.

## 3. Discussion

As one of the most threatening environmental stress factors, salt-alkali stress can hinder plant growth and development in several ways. Accelerated NGS technology enables the investigation of a wide range of physiological and metabolic mechanisms of stress responses at the molecular level. In this study, we first conducted an integrative analysis of mRNA and miRNA expression levels in tobacco under salt and alkali stress. From this integrative analysis, we obtained a valuable set of salt-alkali-responsive mRNAs and miRNAs and identified the interactions and potential roles of these mRNAs and miRNAs in biophysiological processes between NaCl and NaHCO_3_ treatments. These data provide a comprehensive understanding of the differences between salt and alkali stress response mechanisms.

### 3.1. mRNA Profiling in Leaves of Tobacco under Salt-Alkali Stress

Under salt-alkali stress, plants can accumulate essential substances for osmotic adjustment and can regulate gene expression levels to maintain ionic homeostasis [38]. Cation/H^+^ antiporters (CHXs) operate as Na^+^/H^+^ antiporters or K^+^/H^+^ antiporters that help mediate biological responses to excess ions. For example, AtCHX17 is strongly induced by salt stress and is involved in K^+^ acquisition [39]. Moreover, an appropriate K^+^:Na^+^ ratio in the cytoplasm is important for salt tolerance in plants. Overexpression of the potassium channel gene *OsKAT1* has been shown to enhance rice Na^+^ tolerance by regulating K^+^/Na^+^ homeostasis [40]. In our study, the expression of three CHXs and AKT 1 significantly changed under SS and AS treatments (Table 4), suggesting that these ion transporters are responsible for tobacco resistance to salt toxicity via enhanced efflux of Na^+^ and absorption of K^+^. In addition, ABC transporters use energy from ATP hydrolysis for nutrient uptake and play an important role in ion homeostasis [41]. In our study, an increased abundance of ABCG11 but suppressed expression of ABCA7 were identified under both the SS and AS treatments (Table 4). Panikashvili et al. demonstrated that AtWBC11 (AtABCG11) is induced by salt and that wbc11 mutants are highly sensitive to salt stress [42], suggesting a potential role for this protein in plant salt tolerance.

Aquaporins (AQPs) are integral membrane proteins that regulate the transport of water and small molecules. In Arabidopsis and *Beta vulgaris*, the transcript abundance of most of the AQPs decreased in response to salt [43,44]. Hu et al. demonstrated that overexpression of the wheat AQP gene TaAQP8 increased salt stress tolerance in tobacco [45]. The expression levels of eight AQP genes in this study were consistent with previous results (Table 4), indicating that salt-alkali stress disrupts the balance of water transport efficiency [46].

Plant exposure to salt-alkali stress often generates ROS, which cause lipid peroxidation and DNA damage [47]. Plants can mediate the removal of ROS via antioxidant defense enzymes, including superoxide dismutase (SOD), catalase (CAT), peroxidase (POD) and glutathione S-transferase (GST) [48]. Our GO enrichment results indicated that DEGs were significantly enriched in the “antioxidant activity” term under both the SS and AS treatments. As expected, our experimental data showed that the expression of most of the ROS-scavenging enzymes, including SOD, CAT, POD, glutathione peroxidase (GR) and GST, was upregulated under salt-alkali treatment (Table 4), indicating that these scavenging enzymes have active functions that provide resistance to salt and alkali stress in tobacco.

**Figure 6:**
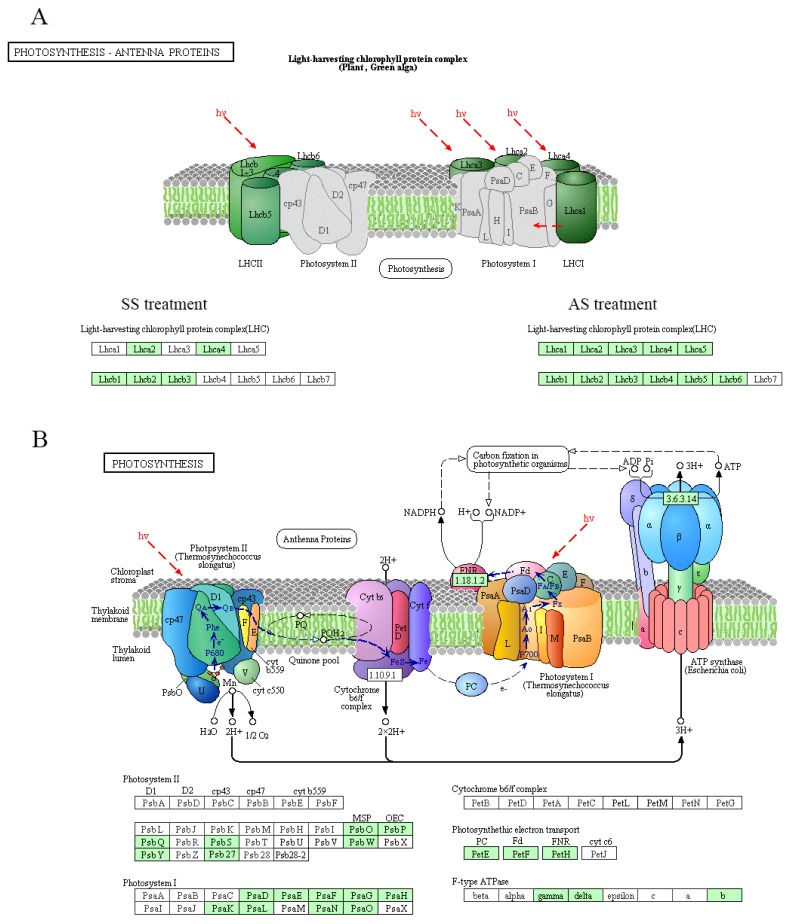
Summary of gene expression related to the photosynthetic machinery in response to SS and AS. KEGG maps of the “photosynthesis-antenna proteins” (**A**) and “photosynthesis” (**B**) pathways are displayed. The green boxes indicate expression levels of the corresponding genes that were downregulated.

Photosynthesis is a well-studied primary metabolic process that is affected by environmental stress. The response of plant photosynthesis to salinity is typically accompanied by stomal closure, reduced CO_2_ diffusion into chloroplasts and alterations in leaf photochemistry [6]. KEGG pathway analysis indicated that DEGs were significantly enriched in “photosynthesis-antenna proteins” under salt-alkali treatment but were enriched in only “photosynthesis” in AS-treated samples. As shown in Figure 6, the transcript abundance of genes involved in the photosynthetic process decreased. Under salt-alkali stress, DEGs encoding light-harvesting complexes, such as Lhca in photosystem I (PSI) and Lhcb in photosystem II (PSII), was downregulated (Figure 6A), suggesting that the capture of light energy was restricted by the SS and AS treatments. Photo-oxidation of water in plants is accomplished by the oxygen-evolving complex of PSII. The decreased abundance of the genes encoding this complex, including PsbO, PsbP, PsbQ, PsbS, PsbY and Psb27, may inhibit the oxygen-evolving capacity of PSII for water oxidation (Figure 6B). Furthermore, the similar expression profiles of DEGs encoding electron transport proteins and ATP synthase indicated an inhibitory effect on the mechanism of electron transport and resulted in the inhibition of ATP synthesis under AS treatment. Taken together, our experimental data revealed that NaHCO_3_ stress strongly affects the process of photosynthesis because of the marked decrease in the expression levels of a wide range of relevant genes.

**Figure 7:**
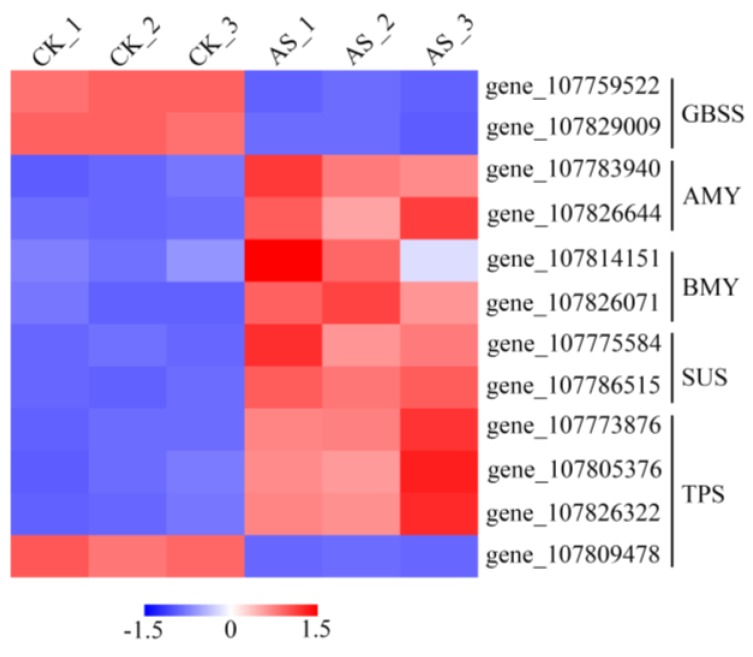
Heatmap of specifically expressed genes related to the “starch and sucrose metabolism” pathway in the AS treatment.

Soluble carbohydrates can promptly accumulate in plants to cope with various forms of abiotic stress. Starch is the predominant carbohydrate storage material that is synthesized by plants. Our KEGG pathway enrichment analysis revealed that the DEGs were markedly enriched in the “starch and sucrose metabolism” pathway under AS treatment but not under SS treatment. Granule-bound starch synthase (GBSS) catalyzes the synthesis of starch, while α-amylase (AMY) and β-amylase (BMY) are responsible for degrading plant starch. Numerous studies have revealed significant changes in the expression levels of GBSS, AMY and BMY under various forms of abiotic stress [49,50]. Similarly, the expression of GBSS 1 was downregulated by NaHCO_3_ treatment, but that of AMY and BMY was upregulated (Figure 7). Zhang et al. reported that the transcript abundance of genes encoding sucrose synthase (Sus) increased under high-pH stress (NaOH treatment). Consistent with this observation, the induction of Sus2 genes by AS in our study may be responsible for high-pH-induced oxygen deficiency conditions, as Sus2 was induced specifically by oxygen deficiency [51]. Trehalose is a non-reducing disaccharide that serves as an energy source for plants subjected to stress [52]. Trehalose-6-phosphate synthase (TPS) is a key enzyme involved in the biosynthesis of trehalose. In our study, we found that the expression of three genes encoding the TPS enzyme was induced and that the expression of only one TPS was downregulated by AS (Figure 7). Taken together, the results show that enhancement of starch and sucrose metabolism may provide energy requirements for tobacco resistance to NaHCO_3_-induced osmotic stress.

### 3.2. miRNA Profiling of the Salt-Alkali Response in Tobacco

Numerous recent studies have demonstrated that miRNAs play crucial roles in plant growth and development and in the plant responses to adverse environmental stress. In our study, we performed sRNA sequencing on the CK, SS and AS samples and identified 89 known miRNAs from 52 families and 102 novel miRNAs. Consistent with the results of a previous sRNA sequencing study in tobacco [33], our results showed that the most abundant transcript was that of nta-miR166a, followed by nta-miR159, nta-miR6149a and nta-miR6021, across the nine libraries (Table S4).

Many miRNAs are closely associated with salt-alkali stress in various plant species, such as Arabidopsis [53], radish [54], flax [55] and *Medicago truncatula* [56]. In the present study, 17 known miRNAs and 16 novel miRNAs were found to respond to salt-alkali stress in tobacco. We detected significant regulation of the miR156, miR482, miR6145 and miR6149 families under both salt and alkali stress. The conserved miR156 family is one of the most well-studied plant miRNA families and plays a vital role in plant stress responses, including salt-alkali resistance in alfalfa [57], cotton [58] and *Populus euphratica* [59]. The roles of miR482 in drought and cold stress, miR6145 in the wound response and miR6149 in Cr stress were also investigated [60,61,62,63]. Our experimental data confirmed the significance of miR156 in tobacco under salt-alkali conditions and provide valuable information concerning the roles of miR482, miR6145 and miR6149 in the response to salt-alkali stress. Notably, two of these overlapping miRNA families, namely, miR156 (nta-miR156a) and miR6145 (nta-miR6145a/e), exhibited differential regulation upon exposure to NaCl and NaHCO_3_ stress in our experiment. A similar result for miR156 was obtained in a previous study, which revealed that the expression of miR156 was downregulated after 4 h [64] but upregulated after 72 h of salt treatment in Medicago [56].

In addition to these four overlapping miRNA families, eight upregulated miRNAs (including nta-miR164a, nta-miR168a, nta-miR168d, nta-miR171c, nta-miR394) and two downregulated miRNAs (nta-miR398, nta-miR408) were markedly expressed under NaHCO_3_ stress, and nta-miR396a was specifically upregulated by NaCl. Liu et al. reported that miR168, miR171 and miR394 were induced and that miR398 was suppressed by salt stress in Arabidopsis [53]. Sun et al. demonstrated that the expression of miR164a and miR396a-3p was upregulated and that the expression of miR398b-3p was downregulated under salt stress in *Raphanus sativus* L. [54]. As miRNAs are evolutionarily conserved between plant species, our work here indicated the occurrence of miRNA regulation in tobacco under salt-alkali stress. Moreover, 16 novel salt-alkali-responsive miRNAs were identified, and the structural precursors of these novel miRNAs are presented in Figure S2. However, further validation is needed to confirm the authenticity of such novel miRNAs accurately.

In general, miRNAs negatively regulate gene transcription levels by target RNA cleavage based on sequence complementarity with targets. Our integrated analysis of miRNA and mRNA transcript levels revealed inverse regulation of 26 and 139 miRNA-mRNA interactions in the SS and AS treatments, respectively. For instance, the miR156 target squamosa promoter-binding protein like (SPL), which plays a role in flower regulation and reproductive growth [65], was also identified in our study. Recently, a role for the miR156/SPL network in the regulation of the content of ROS and salicylic acid (SA) signal transduction was identified in Arabidopsis [66]. As expected, nt-miR156a-targeted SPL members exhibited strict negative regulation in both the SS and AS treatments, suggesting that nt-miR156a plays an important role in salt-alkali stress by regulating the expression of SPLs. Nucleotide-binding sites and leucine-rich repeat (NBS-LRR) regions constitute the main components of disease resistance proteins [67]. These NBS-LRR proteins function in the plant response to pathogenic infection. Interestingly, nt-miR482b-targeted NBS-LRR proteins were identified in our AS treatment, suggesting that this previously verified regulatory mode may also be involved in alkali stress because plants can employ multiple stress response pathways to cope with environmental stress [68,69,70].

Additionally, a large number of salt or alkali stress-responsive DE miRNAs and their targets, such as nta-miR396a/U-BOX, nta-miR408/NAC domain, nta-miR482b-3p/NB-ARC domain, nta-miR6145e/leucine-rich repeat protein kinase, nta-miR6149a/F-BOX and miR-novel_93/Dof domain protein, which may provide salt and alkali stress tolerance, were predicted in our study (Table S5); however, the interactions in this regulatory network need to be further studied.

## 4. Materials and Methods

### 4.1. Plant Materials and Salt-Alkali Treatment

*N. tabacum* ecotype K326 used in this study was obtained from Henan Agricultural University. Tobacco seeds were first sterilized in 75% ethanol for 10 min, rinsed with distilled water three times and then transferred to Murashige and Skoog (MS) medium. After approximately two weeks, germinated seedlings in the MS medium were transferred to plastic pots filled with Hoagland nutrient solution and cultivated in a phytotron under normal conditions (26 °C with 14 h days, 24 °C with 8 h nights, and 70% relative humidity). Tobacco plants grown to the five-leaf stage were used in this study. For salt stress treatment, seedlings were transferred to distilled water supplemented with 100 mM NaCl (pH = 7.0). For alkali stress, seedlings were transferred to distilled water supplemented with 100 mM NaHCO_3_ (pH = 8.4). After they were exposed to the stress treatment for 24 h, the samples were harvested, frozen in liquid nitrogen and then stored at −80°C.

### 4.2. Construction and Sequencing of mRNA-seq and sRNA Libraries

Total RNA was extracted from tobacco leaves using TRIzol reagent (Invitrogen, Carlsbad, CA, USA). The concentration and quality of the RNA samples were assessed using a Nanodrop 2000 instrument (Thermo, Waltham, MA, USA). mRNA-seq libraries were established using an Illumina TruSeq RNA Sample Prep Kit (Illumina, San Diego, CA, USA) according to the manufacturer’s protocol [71]. Briefly, 5 μg of mRNA was isolated from the total RNA using oligo (dT) magnetic beads (Invitrogen, Carlsbadcity, CA, USA), fragmented and reverse transcribed into cDNA. Adapters with a hairpin loop structure were ligated to cDNA molecules and amplified by PCR. The RNA-seq library was sequenced using the Illumina HiSeq X Ten platform (Illumina, San Diego, CA, USA). For small RNA (sRNA) sequencing, RNA bands of approximately 18–30 nt were isolated, linked to 5′ and 3′ adapters and reverse transcribed to cDNA. The final PCR products were purified and sequenced with the Illumina HiSeq 2500 sequencing platform (Illumina, San Diego, CA, USA) with 50-bp single-end reads.

### 4.3. Mapping of mRNA-seq Reads

To obtain clean reads, adapters and low-quality sequence reads were removed from the raw data using Trimmomatic (version 0.30) [72]. These clean reads were mapped to the tobacco genome (available online: ftp://ftp.solgenomics.net/genomes/Nicotiana_tabacum/) using HISAT2 (version 2.0.5) [73]. The gene expression levels in all nine libraries were presented as fragments per kilobase of transcript per million (FPKM) [74].

### 4.4. Analysis of Differential Gene Expression in Response to Salt-Alkali Stress

DEG analysis was carried out using the DESeq R package (1.10.1). Using a model based on the negative binomial distribution, this tool provides statistical routines for determining differential expression within digital gene expression data. On the basis of this method, genes with |log_2_FC|>2 and a *p*-value < 0.05 were classified as DEGs between two comparisons among the three experimental conditions.

### 4.5. GO and KEGG Pathway Enrichment Analysis

GO enrichment analysis of the DEGs was carried out using the GOseq R package with the default parameters, by which gene length bias was corrected [75]. The KEGG database is a widely used resource for understanding high-level functions and utilities of biological systems (available online: http://www.genome.jp/kegg/). The DEGs in the KEGG pathways were analyzed using KOBAS (version 2.0) [76].

### 4.6. Identification of Known and Novel miRNAs and Prediction of miRNA Targets

After the adapter sequences, poly-A sequences and low-quality bases were removed from the raw reads, the remaining unique sequences were mapped to the tobacco genome using Bowtie2 (version 2.0.5) [73]. The sRNA sequences that matched those of rRNA, tRNA, small nuclear RNA (snRNA) and small nucleolar RNA (snoRNA) were excluded. The remaining unique sequences were searched against miRBase 20.0 to identify known miRNAs in tobacco. The software programs miREvo [77] and mirdeep2 [78] were used to predict novel miRNAs and to explore secondary structures, and the miRNA targets were predicted using psRobot [79].

### 4.7. Detection of the Differential Expression of miRNAs in Response to Salt-Alkali Stress

The evaluation of the differential expression of miRNAs between two comparable groups was based on a previously established model [80]. Using |log_2_FC|>0.5 and *p*-value < 0.05 as criteria, we adjusted for multiple testing to classify the DE miRNAs among the three experimental conditions.

### 4.8. Validation of mRNA and miRNA Expression

Total RNA was extracted from each treatment using TRIzol reagent (Invitrogen, USA) as described above. cDNA was generated using a PrimeScript cDNA Synthesis Kit (Takara, Dalian, China) according to the manufacturer’s instructions and then diluted 10 times for use as a qPCR template. sRNA was extracted using a Small RNA Isolation Kit (Takara, Dalian, China). RT-qPCR was performed using SYBR Green Supermix (Takara, Dalian, China) on an ABI StepOne instrument. The tobacco ef1α gene was used as an internal control for mRNA analysis, and 5.8S rRNA was used as the reference for stem-loop miRNA analysis. Three independent biological replicates were included in the qRT-PCR analyses. The relative expression level of each sample was calculated using the 2^−ΔΔ*C*T^ method [81]. The primers used for this RT-qPCR experiment are listed in Table S6.

## 5. Conclusions

In the present study, we comprehensively analyzed the changes in the mRNA and sRNA profiles of tobacco under NaCl and NaHCO_3_ treatment. Overall, 8064 mRNAs and 33 miRNAs were differentially expressed in response to salt and alkali stress. The overlapping DEGs between the two treatments suggested that plants could utilize similar strategies, such as ion channels, AQPs and antioxidant activity, to adapt to both NaCl and NaHCO_3_ stress. However, our GO and KEGG pathway enrichments revealed that many essential biological processes, including “photosynthesis” and “starch and sucrose metabolism,” were specifically enriched in the NaHCO_3_ treatment. In addition, our analysis also revealed many miRNAs, including four overlapping miRNA families, that might play important roles in the response of tobacco to salt and alkali stress. Several inverse regulatory processes of miRNAs with their target transcript genes were also predicted for manipulation. These findings improve our understanding of the underlying salt and alkali responses in tobacco at the molecular level and provide a valuable foundation for future uses of the miRNA-based genetic regulatory network in breeding for salinity tolerance in crops.

## Figures and Tables

**Table 1:** Summary of mRNA sequencing datasets.

Treatment	Raw Reads	Clean Reads	Clean Bases	Q30 (%)	Mapped Reads
CK_1	55,946,150	54,753,170	8.21 G	93.32	51,560,453 (94.17%)
CK_2	52,426,666	50,127,414	7.52 G	93.82	47,158,934 (94.08%)
CK_3	43,620,248	42,722,964	6.41 G	93.71	40,181,708 (94.05%)
SS_1	51,641,948	50,378,090	7.56 G	93.54	47,605,747 (94.5%)
SS_2	61,792,742	60,177,334	9.03 G	93.74	56,855,737 (94.48%)
SS_3	65,172,280	64,106,196	9.62 G	93.64	60,509,819 (94.39%)
AS_1	64,693,340	63,648,474	9.55 G	93.49	59,519,456 (93.51%)
AS_2	58,326,842	57,264,752	8.59 G	93.49	53,875,061 (94.08%)
AS_3	57,919,408	57,037,200	8.56 G	93.24	53,759,723 (94.25%)

**Table 2:** Summary of small RNA (sRNA) sequencing datasets.

Treatment	Raw Reads	Clean Reads	Mature miRNAs	Novel miRNAs
CK_1	15,192,456	14,706,127	79	99
CK_2	16,160,064	15,672,009	74	99
CK_3	13,289,928	12,949,487	67	99
SS_1	13,583,796	13,079,201	80	100
SS_2	12,296,168	11,973,197	72	97
SS_3	14,031,804	13,667,590	76	100
AS_1	13,551,741	13,090,227	80	99
AS_2	15,333,030	14,915,331	78	98
AS_3	13,272,983	12,863,432	81	101

**Table 3:** List of differentially expressed microRNAs (DE miRNAs) in response to SS and AS treatments.

miRNA	log_2_FC	*p*-Value	Mature Sequence	Regulated
nta-miR156a	−0.64	2.56 × 10^−2^	UGACAGAAGAGAGUGAGCAC	SS
nta-miR396a	0.64	1.07 × 10^−3^	UUCCACAGCUUUCUUGAACUG	SS
nta-miR482d	0.78	4.42 × 10^−4^	UUCCCGACUCCCCCCAUACCAC	SS
nta-miR6145a	−0.55	4.91 × 10^−2^	CAUUUUCACAUGUAGCACUGAC	SS
nta-miR6149a	0.75	1.51 × 10^−3^	UUGAUACGCACCUGAAUCGGC	SS
miR-novel_31	−0.93	4.56 × 10^−4^	AAGGACUGCUAUUUAGAAAUUAGC	SS
miR-novel_59	−0.57	2.21 × 10^−2^	ACGGGCUGCUAUUUAAGAAUUAGC	SS
miR-novel_61	1.15	1.82 × 10^−5^	UUGAAGUGUUUGGGGGAACUC	SS
miR-novel_64	−0.98	1.62 × 10^−3^	CUCCGGUCAAGAUCUUCCAUC	SS
miR-novel_127	−0.71	3.30 × 10^−2^	ACGUGUCGGAUCCUCUAAAAGU	SS
miR-novel_163	−0.81	1.48 × 10^−2^	AUGGCGUGGCUUCAGAUCUCUGAU	SS
miR-novel_216	−1.86	3.39 × 10^−8^	AGGACGAUCUCUAUGACUCUUUGG	SS
miR-novel_228	−0.88	9.24 × 10^−3^	ACGUGGACUGGGCUGGGCUAGCCU	SS
miR-novel_237	−0.65	3.14 × 10^−2^	AGGACUGCUAUUUAGAAAUUAGGC	SS
miR-novel_258	−0.84	7.63 × 10^−3^	ACUCCGAUUUGUGUAUGACUUGAC	SS
nta-miR156a	0.71	2.82 × 10^−2^	UGACAGAAGAGAGUGAGCAC	AS
nta-miR164a	0.84	2.40 × 10^−2^	UGGAGAAGCAGGGCACGUGCA	AS
nta-miR168a	0.83	3.65 × 10^−4^	UCGCUUGGUGCAGGUCGGGAC	AS
nta-miR168d	0.73	2.59 × 10^−2^	UCGCUUGGUGCAGGUCGGGAA	AS
nta-miR171c	1.00	8.94 × 10^−3^	UGAUUGAGCCGUGCCAAUAUC	AS
nta-miR394	0.85	1.83 × 10^−2^	UUGGCAUUCUGUCCACCUCC	AS
nta-miR398	−0.70	2.68 × 10^−2^	UGUGUUCUCAGGUCGCCCCUG	AS
nta-miR408	−0.70	1.94 × 10^−2^	UGCACUGCCUCUUCCCUGGCU	AS
nta-miR482b-3p	0.85	1.61 × 10^−2^	UCUUGCCAAUGCCAUCCAUUCC	AS
nta-miR6020b	1.12	2.31 × 10^−3^	AAAUGUUCUUCGAGUAUCUUC	AS
nta-miR6145e	0.54	4.90 × 10^−2^	AUUGUUACAUGUAGCACUGGC	AS
nta-miR6149a	0.69	2.27 × 10^−2^	UUGAUACGCACCUGAAUCGGC	AS
nta-miR6159	0.72	1.57 × 10^−2^	UAGCAUAGAAUUCUCGCACCUA	AS
nta-miR827	0.64	3.92 × 10^−2^	UUAGAUGAACAUCAACAAACA	AS
miR-novel_43	−1.08	5.83 × 10^−5^	UUGGGCGUGCACAAGUAGGC	AS
miR-novel_64	−1.17	1.38 × 10^−3^	CUCCGGUCAAGAUCUUCCAUC	AS
miR-novel_93	1.18	1.42 × 10^−3^	ACGCAGGAGAGAUGAUGCUGGA	AS
miR-novel_156	−0.91	1.26 × 10^−2^	ACCGAUGUGGGACUCUGUCUAAAG	AS
miR-novel_167	−0.91	9.95 × 10^−3^	AAAGAGCUCUAUAUAUAAAAAUGU	AS
miR-novel_193	−0.81	2.60 × 10^−2^	AAGUCCGGUAACAUUUUGAAGAGU	AS
miR-novel_215	−1.29	1.11 × 10^−4^	ACGUGGGCUGGGCUGAGCUAACCC	AS
miR-novel_216	−1.31	4.12 × 10^−4^	AGGACGAUCUCUAUGACUCUUUGG	AS

**Table 4:** Candidate DEGs under SS and AS treatment.

Gene ID	CK (FPKM)	SS (FPKM)	AS (FPKM)	Log_2_FC (SS/CK)	Log_2_FC (AS/CK)	Regulation	Annotation
Ion transport							
107775281	0.13	0	0	−inf	−inf	Down	cation/H(+) antiporter 18
107803899	0.02	0.18	0.13	3.05	2.46	Up	cation/H(+) antiporter 15
107814619	0.02	0.19	0.67	2.83	4.61	Up	potassium channel AKT1
107774409	0.04	0.28	9.04	2.68	7.62	Up	ABC transporter G family member 11
107800765	1.53	0.40	0.20	−2.22	−3.26	Down	ABC transporter B family member 19
Aquaporins							
107762778	12.65	1.79	1.38	−3.12	−3.56	Down	aquaporin PIP2-1
107782948	42.89	6.99	6.57	−2.92	−3.07	Down	aquaporin PIP2-1
107786743	39.82	7.32	4.89	−2.74	−3.39	Down	aquaporin PIP2-7
107789972	95.21	15.78	12.69	−2.89	−3.27	Down	aquaporin PIP2-7
107807226	75.86	8.04	6.26	−3.54	−3.96	Down	aquaporin TIP1-3
107814785	34.86	2.09	2.89	−4.36	−3.95	Down	aquaporin TIP2-1
107820018	31.08	7.70	6.70	−2.31	−2.58	Down	aquaporin TIP1-3
107821662	4.55	1.28	0.39	−2.13	−3.92	Down	aquaporin TIP2-1
Antioxidant activity							
107819573	0.08	0.48	0.71	2.23	2.74	Up	superoxide dismutase family
107832827	0.25	1.71	2.38	2.45	2.86	Up	superoxide dismutase family
107781013	2.73	17.09	16.50	2.34	2.23	Up	catalase family
107758994	1.20	0.11	0	−3.74	−inf	Down	peroxidase 19
107766849	0.28	0	0	−inf	−inf	Down	peroxidase 10
107777661	0.40	3.98	2.17	3.04	2.08	Up	peroxidase P7
107793453	0.48	3.42	5.06	2.56	3.05	Up	peroxidase P7
107813838	0.24	2.62	1.34	3.17	2.13	Up	peroxidase 12
107814038	0.14	0	0	−inf	−inf	Down	peroxidase 3
107820597	0.81	4.34	5.04	2.13	2.28	Up	probable glutathione peroxidase 8
107777621	6.99	40.69	87.80	2.24	3.29	Up	glutathione transferase GST 23
107764067	1.12	14.98	74.03	3.44	5.68	Up	glutathione transferase GST 23

## Supplementary Materials

Supplementary File 1. Supplementary materials can be found at https://www.mdpi.com/1422-0067/20/10/2391/s1.
