# Supplementary figures and images for: Integration of mRNA and miRNA Analysis Reveals the Molecular Mechanism Underlying Salt and Alkali Stress Tolerance in Tobacco
Source: Int J Mol Sci. 2019 May 14;20(10):2391. doi: 10.3390/ijms20102391 (PMC6566703; doi:10.3390/ijms20102391)

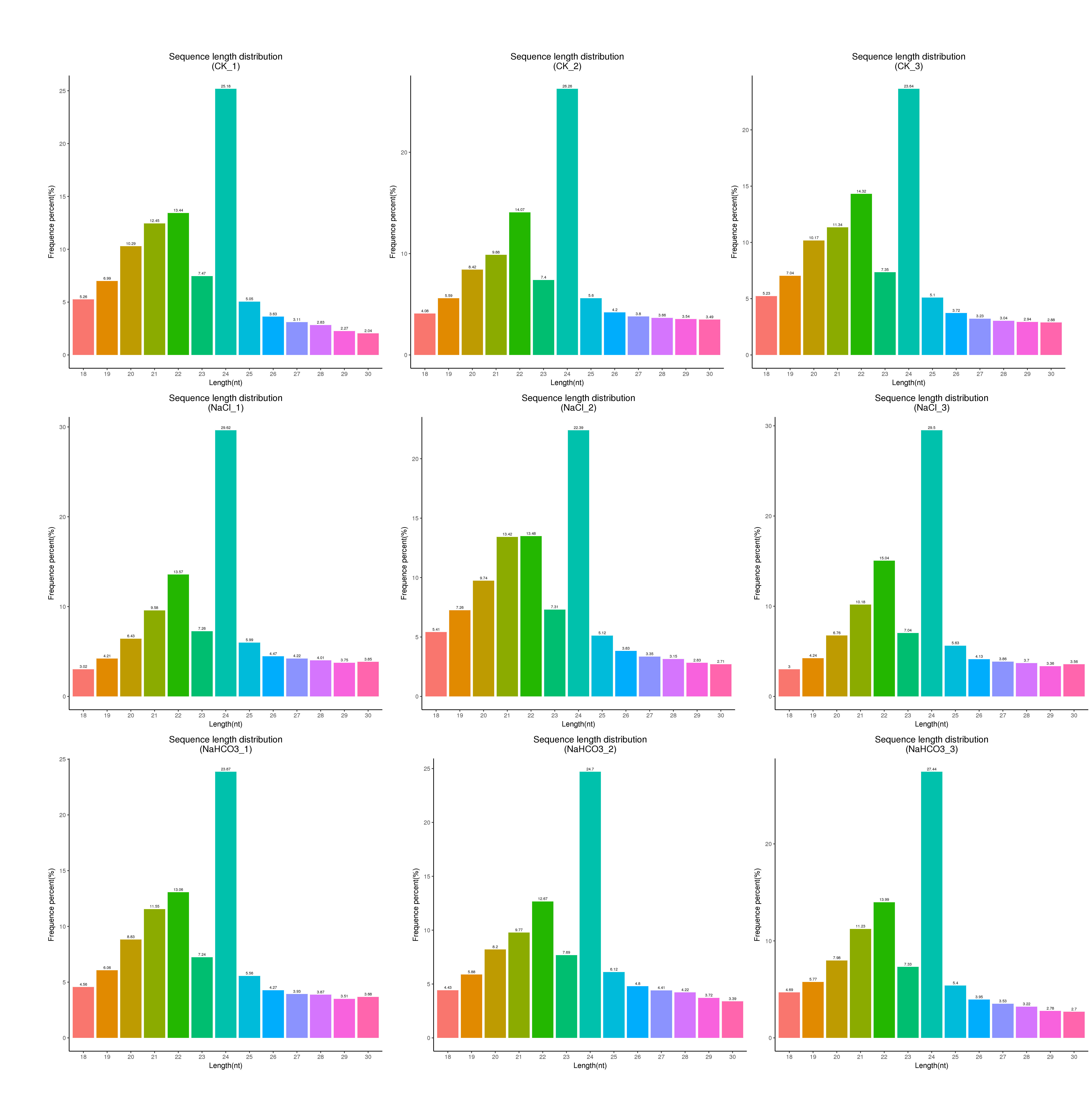

Supplement: Supplementary file 1 [file ijms-20-02391-s001.zip › Supplementary File 1/Fig S1.tif]
